# Design and Modeling of a 2-DOF Micro-Positioning Stage for Vibration-Assisted Polishing

**DOI:** 10.3390/mi16111250

**Published:** 2025-10-31

**Authors:** Panpan Chen, Yangmin Li

**Affiliations:** 1 School of Engineering, Shandong Xiehe University, Jinan 250109, China; panpanchen29@hotmail.com; 2 Faculty of Data Science, City University of Macau, Taipa, Macau 999078, China; 3 Department of Industrial & Systems Engineering, The Hongkong Polytechnic University, Hong Kong 999077, China

**Keywords:** vibration-assisted polishing, compliant precision stage, amplification mechanism, Castigliano’s second theorem, elastic beam theory

## Abstract

To solve the issues of insufficient working stroke, low accuracy, and limited response time of stages for vibration-assisted polishing, a two-degree-of-freedom (2-DOF) micro-positioning stage is proposed in this paper. To compensate for the limited stroke of piezoelectric actuator, a bridge–lever amplification mechanism was designed to magnify output displacement. Based on Castigliano’s second theorem and elastic beam theory, static modeling of amplification mechanisms, guiding beams, and transmitting rods was presented. Then, the analytical models of the stage were derived. To validate the accuracy of the analytical model, finite element simulations were performed, demonstrating that the error between theoretical and simulation results is 4.6%. Notably, the stage exhibits kinematic decoupling characteristics and excellent dynamic performances. The research results can provide effective insights for developing a large-stroke piezo-actuated micro-positioning stage with good dynamic performance for vibration-assisted polishing.

## 1. Introduction

High-precision optical components are widely used in high-precision systems, such as mirror glass for space telescopes and biomedical imaging instruments [1]. However, the surface of optical components is easily damaged and worn out due to inherent brittle materials; thus grinding and polishing the surface is necessary [2,3,4,5]. In order to elevate manufacturing precision and efficiency, vibration-assisted mechanisms are incorporated into polishing systems [6,7,8,9].

Vibration-assisted polishing can improve the surface quality of processed components due to its low polishing force, low grinding speed, and high vibration frequency [10,11,12]. This approach only works at a resonant frequency, allowing for efficient utilization of the mechanical vibration energy. Compared with resonant mode, polishing systems using piezo-actuated stages can exhibit superior vibration characteristics in non-resonant mode, which has inherent merits of adjustable frequencies and precise motion [13]. For instance, Zhao et al. investigated an ultrasonic vibration-assisted polishing mechanism, which features high vibration frequency and amplitude, resulting in a lower surface roughness and less polishing marks of the microcylindrical surface on SiC [4]. Lin et al. proposed a new vibration device for multi-angle ultrasonic polishing of complex surfaces of biomaterials [12]. The conducted experiments demonstrate that the surface roughness of biomaterials can be reduced from 235 nm to 140 nm in comparison to conventional mechanical polishing. Gu et al. designed a two-dimensional vibration-assisted machine for polishing SiC ceramics [14]. The experimental results have shown that the proposed device can machine hard and brittle materials. Additionally, Jia et al. utilized multi-angle two-dimensional ultrasonic vibration on zirconia ceramic grinding via a combination of nanofluid minimum quantity lubrication and ultrasonic machining [15]. The research results reveal that adhesion and material peeling can be effectively reduced.

Compared to conventional rigid mechanisms, compliant mechanisms have obvious superiority, e.g., no backlash, no wear, high-precision motion, etc. [16,17,18,19,20]. To date, vibration-assisted polishing technologies based on high-precision compliant stages have been widely developed to achieve precise, reliable motion at the micro- or nano-scale [21,22,23,24]. For instance, Chee et al. proposed a two-dimensional low-frequency vibration polishing mechanism using piezoelectric actuators [25]. The polishing experiments show that it can achieve vibration in a circle trajectory to assist with polishing. Furthermore, Chee et al. developed a novel polishing system for micro-molds, which can realize low-contact-force polishing of the micro-molds through two-dimensional low-frequency vibration and a mechanical transformer mechanism [26]. Guo et al. explored effects of process parameters on material removal in vibration-assisted polishing, revealing that a 2D vibrating motion can create better surface roughness with higher material removal efficiency [27]. However, piezoelectric actuators have a small output stroke, resulting in limitations to the micro-positioning stage in precision operation applications [28,29,30,31].

To address this issue, a previous literature review has shown that various studies on micro-positioning stages focus on configuration design of the precision stage with a displacement amplifier [17]. Some typical amplification mechanisms have been studied, e.g., lever mechanism [32], bridge-type mechanism [33], Scott Russell mechanism [34], triangular mechanism [35], rhombus mechanism [36], and hybrid mechanism [37]. For instance, Gu et al. also proposed a 2-DOF flexure-based vibration-assisted polishing device based on a two-level lever amplification mechanism [13]. For realizing vibration-assisted roller polishing on silicon carbides, Gu et al. designed a 2-DOF decoupling piezo-actuated device using lever amplification, with a working space of 38 µm × 42 µm and natural frequency of 1198 Hz [23]. Van et al. adopted three-lever amplification mechanisms and parallelograms to improve working stroke and resonant frequency [24]. The experimental results show that the resonant frequency of the 2-DOF precision stage is about 486 Hz, exhibiting a potential application in vibration-assisted polishing. Gu et al. developed a 2-DOF piezo-driven compliant micro-motion stage for a vibration-assisted roll-type polishing system, which can obtain a high-quality surface using silicon carbide ceramic with 35 nm Sa, as shown by experimental validation [38].

The objective of the paper is to present a 2-DOF piezo-driven precision motion stage with a promising application in vibration-assisted polishing. The bridge–lever hybrid mechanisms are employed for amplifying the output displacement of piezoelectric actuators. The multi-stage parallelogram guiding mechanism and series-parallel chain are used for obtaining a wide range of frequencies. The static and dynamic performance of the motion stage is developed using Castigliano’s second theorem, elastic beam theory, and the Lagrange equation. The validation of analytical findings is supported by simulation calculation.

The rest of the paper is arranged as follows: Section 2 presents the design of the 2-DOF flexure-based micro-positioning stage. Section 3 presents the static model and parametric analysis of the compliant stage. Section 4 presents the dynamic modeling analysis of the compliant stage. Section 5 presents the simulation analysis of the compliant stage. Section 6 presents the conclusions.

## 2. Mechanism Design

**Figure 1:**
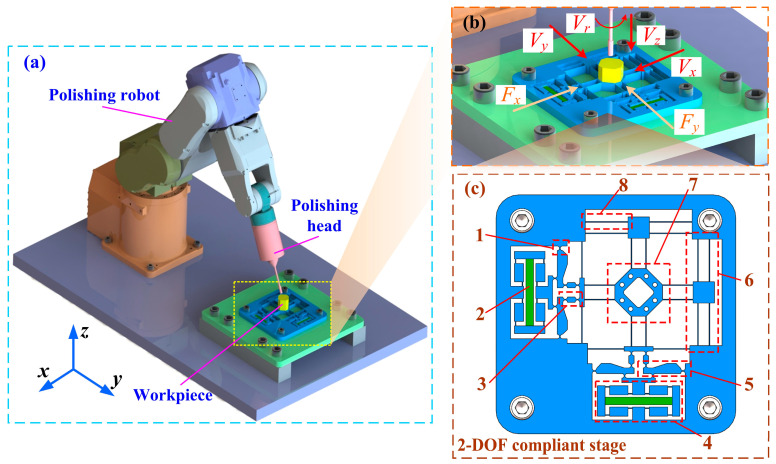
Vibration-assisted polishing system using piezo-actuated compliant micro-positioning stages. (**a**) System structure. (**b**) Vibration-assisted polishing principle. (**c**) 2-DOF stage: 1—circular hinge; 2—piezoelectric actuator; 3—transmitting mechanism; 4—bridge mechanism; 5—lever mechanism; 6—guiding mechanism; 7—working platform; 8—beam hinge.

Figure 1a depicts the vibration-assisted polishing system which integrates with a 2-DOF compliant stage. The working principle of the system is that the 2-DOF positioner generates small-amplitude motion with a high frequency by a controlled piezoelectric actuator when the polishing head executes polishing operations. Figure 1c shows the configuration of the proposed micro-positioner, which includes a hybrid bridge–lever amplification mechanism to compensate for the stroke of the piezoelectric actuator. The bridge–lever mechanism employs a series method for motion transmission, in which initial input displacement from piezoelectric actuator is magnified in the motion of the ending platform. When the piezoelectric actuator generates displacement at two input ends of the bridge mechanism, the output displacement is magnified due to the triangle principle and acts at the input-end of the second-level lever mechanism. With the action of the driving force from the bridge mechanism, the output displacement is amplified due to the lever principle and finally acts at the working platform via a transmitting mechanism. Notably, guiding-motion mechanisms are utilized to eliminate parasitic displacement for ensuring precise translational motion. To balance stiffness and working displacement, beam flexures are used for bridge mechanisms and guiding mechanisms. On the other hand, circular hinges, known for precise motion without drift, are utilized for lever mechanisms [39]. Furthermore, for minimizing bending deformation of the lever during operation and enhancing its response speed, the lever mechanism is designed with a specific geometric configuration [40].

## 3. Static Modeling Analysis

In this Section, we present the analytic model and static analysis of the compliant stage.

### 3.1. Static Modeling of Bridge Mechanism

**Figure 2:**
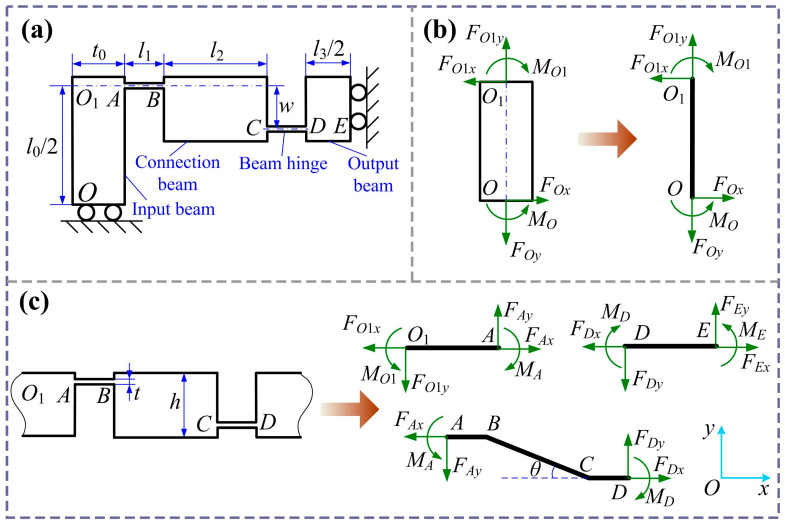
Schematic of the bridge amplification mechanism: (**a**) 1/4 model; (**b**) model of input beam; (**c**) model of flexure hinge, connecting beam, and output beam.

Figure 2 shows a quarter of the bridge mechanism. *l*_0_ and *t*_0_ are the length and width of the beam *OO*_1_, *l*_2_ and *h* denote the length and width of the connection beam BC, *l*_3_ is the length of the beam DE, *l*_1_ and *t* are the length and width of the straight beam’s hinge, and *w* is the horizontal distance between the two hinges. For the input beam, the force and moment balance can be expressed as shown below the figure.

(1) $$F_{ox}=F_{o1x}=F_{x}=\frac{1}{2}F_{in}$$

(2) $$F_{oy}=F_{o1y}=F_{y}=\frac{1}{2}F_{out}$$

(3) $$M_{o1}=M_{O}-F_{x}\cdot \frac{l_{0}}{2}$$

where *F_in_* and *F_out_* denote the input force and output force of the bridge amplification mechanism. Considering the same deformation of flexure hinges AB and CD, and angle displacement of zero at points A and D, to simplify the calculation process, it is assumed that *M_A_* = *M_D_* = *M*. The bending moments for the flexible hinges, connection beam, and output beam are calculated as follows:

(4) $$M_{A}+M_{D}=2M=F_{x}\cdot w-F_{y}\cdot (2l_{1}+l_{2})$$

(5) $$M_{AB}(x)=M+F_{y}\cdot x$$

(6) $$M_{BC}(x)=M+F_{y}\cdot x-F_{x}\cdot (x-l_{1})\cdot \tan\theta$$

(7) $$M_{CD}(x)=M+F_{y}\cdot x-F_{x}\cdot l_{2}\cdot \tan\theta$$

(8) $$M_{DE}(x)=M-F_{y}\cdot (x-2l_{1}-l_{2})$$

The moment *M* is expressed by *F_x_* and *F_y_*, and the following relationships can be derived:

(9) $$M=\frac{1}{2}[F_{x}\cdot w-F_{y}\cdot (2l_{1}+l_{2})]$$

Note that $\tan\theta =w/l_{2}$, according to Equation (9); Equations (7) and (8) can be written as follows:

(10) $$M_{AB}(x)=\frac{1}{2}\left[w\cdot F_{x}-(2l_{1}+l_{2}-2x)\cdot F_{y}\right](0\leq x\leq l_{1})$$

(11) $$M_{BC}(x)=\frac{1}{2}\left[w-2(x-l_{1})\cdot \frac{w}{l_{2}}\right]\cdot F_{x}-\frac{1}{2}(2l_{1}+l_{2}-2x)\cdot F_{y}(l_{1}\leq x\leq l_{1}+l_{2})$$

(12) $$M_{CD}(x)=-\frac{1}{2}w\cdot F_{x}-\frac{1}{2}(2l_{1}+l_{2}-2x)\cdot F_{y}(l_{1}+l_{2}\leq x\leq 2l_{1}+l_{2})$$

(13) $$M_{DE}(x)=\frac{1}{2}w\cdot F_{x}-\frac{1}{2}(2x-2l_{1}-l_{2})\cdot F_{y}(2l_{1}+l_{2}\leq x\leq 2l_{1}+l_{2}+\frac{l_{3}}{2})$$

The bending moment at point *O*_1_ can expressed as follows:

(14) $$M_{O1}=M_{A}-F_{y}\cdot \frac{t_{0}}{2}=M-F_{y}\cdot \frac{t_{0}}{2}$$

Substituting Equation (9) into Equation (14), we can obtain

(15) $$M_{OO_{1}}(y)=\frac{1}{2}(w+l_{0}-2y)\cdot F_{x}-\frac{1}{2}(2l_{1}+l_{2}+t_{0})\cdot F_{y}(0\leq y\leq \frac{1}{2}l_{0})$$

The input and output displacement at points *O* and *D* can be determined through Castigliano’s second theorem. In general, the fixed end is considered to be rigid, and the remaining parts are regarded as flexible. Therefore, the input and output displacements of the bridge mechanism can be expressed as follows:

(16) $$\left(\begin{array}{c} x_{in}\\ x_{out} \end{array}\right)=\left[\begin{array}{cc} C_{11} & C_{12}\\ C_{21} & C_{22} \end{array}\right]\cdot \left(\begin{array}{c} F_{x}\\ F_{y} \end{array}\right)$$

where $\left[\begin{array}{cc} C_{11} & C_{12}\\ C_{21} & C_{22} \end{array}\right]$ is compliance matrix, which is expressed as follows:

(17) $$\left\{\begin{array}{l} C_{11}=\frac{2}{k_{l1}}+\frac{1}{k_{l2}}+\frac{1}{k_{l3}}+\frac{1}{4k_{\theta 0}}\left(w^{2}+wl_{0}+\frac{1}{3}l_{0}^{2}\right)+\frac{w^{2}}{2k_{\theta 1}}+\frac{w^{2}}{12k_{\theta 2}}+\frac{w^{2}}{4k_{\theta 3}}\\ C_{12}=\frac{1}{4k_{\theta 0}}\left(w+\frac{1}{2}l_{0}\right)(2l_{1}+l_{2}+t_{0})+\frac{w(l_{1}+l_{2})}{2k_{\theta 1}}+\frac{wl_{2}}{12k_{\theta 2}}+\frac{w}{4k_{\theta 3}}\left(2l_{1}+l_{2}+\frac{1}{2}l_{3}\right)\\ C_{21}=\frac{1}{4k_{\theta 0}}\left(w+\frac{1}{2}l_{0}\right)(2l_{1}+l_{2}+t_{0})+\frac{w(l_{1}+l_{2})}{2k_{\theta 1}}+\frac{wl_{2}}{12k_{\theta 2}}+\frac{w}{4k_{\theta 3}}\left(2l_{1}+l_{2}+\frac{1}{2}l_{3}\right)\\ C_{22}=\frac{1}{k_{10}}+\frac{{\left(2l_{1}+l_{2}+t_{0}\right)}^{2}}{4k_{\theta 0}}+\frac{1}{2k_{\theta 1}}\left(\frac{4}{3}l_{1}^{2}+2l_{1}l_{2}+l_{2}^{2}\right)+\frac{l_{2}^{2}}{12k_{\theta 2}}+\frac{1}{4k_{\theta 3}}\left[{\left(2l_{1}+l_{2}\right)}^{2}+l_{3}(2l_{1}+l_{2})+\frac{1}{3}l_{3}^{2}\right] \end{array}\right.$$

where $k_{l0}$, $k_{l1}$, $k_{l2}$, and $k_{l3}$ are axial tensile stiffness of the input beam, beam flexible hinges, connection beam, and output beam, respectively. $k_{\theta 0}$, $k_{\theta 1}$, $k_{\theta 2}$, and $k_{\theta 3}$ are the rotational stiffness of the input beam, beam flexible hinges, connection beam, and output beam, respectively. Considering the plane stiffness of the bridge amplification mechanism [41,42,43], the following results are obtained:

(18) $$\left\{\begin{array}{c} k_{l_{0}}=\frac{2Ebt_{0}}{l_{0}},k_{l_{1}}=\frac{Ebt}{l_{1}},k_{l_{2}}=\frac{Ebh}{l_{2}},k_{l_{3}}=\frac{2Ebh}{l_{3}}\\ k_{\theta_{0}}=\frac{Ebt_{0}^{3}}{6l_{0}},k_{\theta_{1}}=\frac{Ebt^{3}}{12l_{1}},k_{\theta_{2}}=\frac{Ebh^{3}}{12l_{2}},k_{\theta_{3}}=\frac{Ebh^{3}}{6l_{3}} \end{array}\right.$$

where *E* is Young’s modulus, and *b* is the out-of-plane width. The amplification ratio and input stiffness of the bridge-type amplification mechanism are obtained:

(19) $$\lambda_{b}=\frac{x_{out}}{x_{in}}=\frac{C_{21}}{C_{11}}$$

(20) $$k_{b\_in}=\frac{1}{C_{11}}$$

When *x_in_* = 0, the output stiffness of the bridge mechanism with loading is written as follows:

(21) $$k_{b\_out}=\frac{C_{11}}{C_{11}C_{22}-C_{12}C_{21}}$$

Since the analytical model is established based on a quarter structure of the bridge mechanism, the overall displacement amplification ratio of the bridge mechanism with two output ends can be derived by

(22) $$\lambda_{B}=2\lambda_{b}=\frac{\frac{1}{2k_{\theta 0}}\left(w+\frac{1}{2}l_{0}\right)(2l_{1}+l_{2}+t_{0})+\frac{w(l_{1}+l_{2})}{k_{\theta 1}}+\frac{wl_{2}}{6k_{\theta 2}}+\frac{w}{2k_{\theta 3}}\left(2l_{1}+l_{2}+\frac{1}{2}l_{3}\right)}{\frac{2}{k_{l1}}+\frac{1}{k_{l2}}+\frac{1}{k_{l3}}+\frac{1}{4k_{\theta 0}}\left(w^{2}+wl_{0}+\frac{1}{3}l_{0}^{2}\right)+\frac{w^{2}}{2k_{\theta 1}}+\frac{w^{2}}{12k_{\theta 2}}+\frac{w^{2}}{4k_{\theta 3}}}$$

### 3.2. Static Modeling of Lever Mechanism

**Figure 3:**
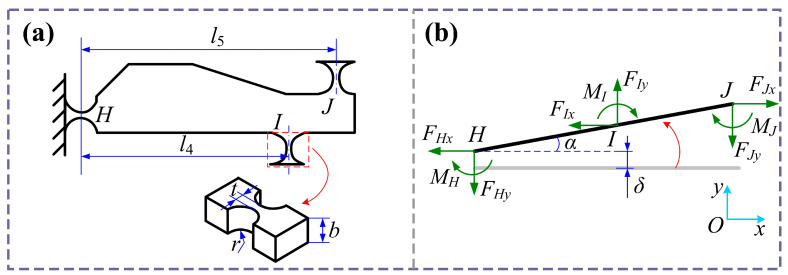
Diagram of lever amplification mechanism. (**a**) Structural architectural parameters. (**b**) Static model.

Figure 3 illustrates the static model of the lever mechanism. In the model, only the deformation caused by lever mechanism motion is considered, while the transmission mechanism is treated as a rigid component. Here, *l*_4_ represents the horizontal distance between hinges *H* and *I*, *l*_5_ is the horizontal distance between hinges *H* and *J*, and *r* and *t* denote the circular radius and minimum width of the circular hinge, respectively. The lever mechanism is subjected to a force from the bridge mechanism, causing the hinge to rotate with an angle *α*. Due to bending of the hinge, the lever component deviates from its initial position, producing a displacement *δ*.

One may observe from Figure 3b that the amplification ratio and stiffness of the lever mechanism are expressed as follows:

(23) $$\lambda_{L}=\frac{l_{5}\alpha +\delta }{l_{4}\alpha +\delta }$$

(24) $$k_{L}=\frac{F_{Iy}}{l_{4}\alpha +\delta }$$

Then, we can obtain

(25) $$F_{Iy}=F_{Hy}+F_{Jy}$$

(26) $$F_{Iy}l_{4}=F_{Jy}l_{5}+M_{H}+M_{I}+M_{J}$$

Additionally,

(27) $$\left\{\begin{array}{c} F_{Hy}=k_{Hb}\delta ,F_{Hy}=k_{Jb}\left(l_{5}\alpha +\delta \right)\\ M_{H}=k_{Ht}\alpha ,M_{I}=k_{It}\alpha ,M_{J}=k_{Jt}\alpha \end{array}\right.$$

where *k_Hb_* and *k_Jb_* are bending stiffness of the flexible hinges *H* and *J*, respectively. *k_Ht_*, *k_It_*, and *k_Jt_* are the torsion stiffness of the flexible hinges *H*, *I*, and *J*, respectively. According to reference [42], the bending stiffness and torsion stiffness of circular hinges in the lever mechanism can be expressed as follows:

(28) $$\left\{\begin{array}{c} k_{Hb}=k_{Jb}=\frac{2Ebt^{2.5}}{3\pi r^{1.5}(3r+t)}\\ k_{Ht}=k_{It}=k_{Jt}=\frac{2Ebt^{2.5}}{9\pi r^{0.5}} \end{array}\right.$$

Substituting Equations (27) and (28) into Equations (25) and (26), we can obtain

(29) $$\alpha =\frac{(k_{Hb}+k_{Jb})l_{4}-k_{Jb}l_{5}}{(k_{Hb}+k_{Jb})(k_{Ht}+k_{Jt})+2k_{Hb}k_{Jb}l_{5}^{2}}F_{Iy}$$

(30) $$\delta =\frac{k_{Ht}+k_{Jt}+k_{Jb}l_{4}^{2}-k_{Jb}l_{3}l_{4}}{(k_{Hb}+k_{Jb})(k_{Ht}+k_{Jt})+2k_{Hb}k_{Jb}l_{4}^{2}}F_{Iy}$$

Substituting Equations (29) and (30) into Equations (23) and (24), the amplification ratio and stiffness of the lever mechanism can further be written as follows:

(31) $$\lambda_{L}=\frac{k_{Hb}l_{4}l_{5}+2k_{Ht}+k_{Jt}}{k_{Hb}l_{4}^{2}+2k_{Ht}+k_{Jt}+k_{Jb}{\left(l_{5}-l_{4}\right)}^{2}}$$

(32) $$k_{L}=\frac{(k_{Hb}+k_{Jb})(2k_{Ht}+k_{Jt})+k_{Hb}k_{Jb}l_{4}^{2}}{k_{Hb}l_{4}^{2}+2k_{Ht}+k_{Jt}+k_{Jb}{\left(l_{5}-l_{4}\right)}^{2}}$$

### 3.3. Static Modeling of Guiding Mechanisms

**Figure 4:**
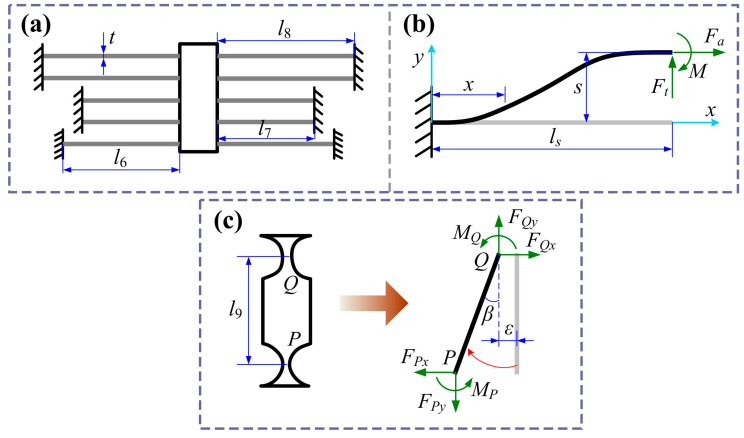
(**a**) The equivalent model of the guiding mechanism. (**b**) Mechanical modeling of the flexible beam. (**c**) Modeling of transmitting mechanism.

The guiding mechanism ensures a purely translational motion of the positioning stage. However, it also increases the stiffness of the amplification mechanism, which reduces the output displacement. Therefore, it is necessary to consider the load effect to improve the accuracy of the theoretical model. The guiding mechanism consists of five pairs of flexible straight beams, and its equivalent model is shown in Figure 4a. Due to the symmetric structure, only a single flexible straight beam needs to be analyzed, as illustrated in Figure 4b.

According to the elastic beam theory [43], the approximate differential equation of the deformation curve for the flexible beam can be expressed as

(33) $$EI_{s}w^{\prime \prime }(x)=M(x)=F_{t}(l_{s}-x)-M$$

where *I_s_* = *bt_s_*^3^/12 is moment of the inertia, *M*(*x*) is the equivalent bending moment of the beam along the *x*-direction, *w*(*x*) is the bending deflection along the *y*-direction, and *F_t_*, *F_a_*, and *M* are generalized forces applied at the endpoint of beam. Parameters *b*, *t_s_*, and *l_s_* represent the width, thickness, and length of the beam, respectively. Then the stiffness of a single beam is expressed as follows:

(34) $$k_{s}=\frac{12EI_{s}}{l_{s}^{3}}$$

The guiding mechanism consists of eight flexible straight beams in parallel, so the equivalent stiffness of the guiding mechanism is

(35) $$k_{g}=\frac{2Ebt^{3}}{l_{6}^{3}}+\frac{4Ebt^{3}}{l_{7}^{3}}+\frac{4Ebt^{3}}{l_{8}^{3}}$$

Except for the guiding mechanism, the transmission mechanism plays a key role in eliminating parasitic motion of the lever mechanism, meanwhile providing a load effect on it. The model of the transmission mechanism is shown in Figure 4c. Here, *l*_9_ represents the vertical distance between hinges *P* and *Q*. When subjected to the output force from the lever mechanism, hinge *Q* is driven to rotate with an angle *β*. Due to the bending of the hinge, an axial drift displacement *ε* is generated. Like the modeling of the lever mechanism, only the angular change in hinge *P* is caused by the transmission mechanism. Thus, the stiffness of the transmission mechanism can be expressed as follows:

(36) $$k_{d}=\frac{F_{Px}}{l_{9}\beta +\varepsilon }$$

Based on force and moment balance equation, we can obtain

(37) $$\left\{\begin{array}{c} F_{Px}=F_{Qx}\\ F_{Px}l_{9}=M_{P}+M_{Q} \end{array}\right.$$

Meanwhile,

(38) $$\left\{\begin{array}{c} F_{Px}=k_{Pb}\left(l_{9}\beta +\varepsilon \right),F_{Qx}=k_{Qb}\varepsilon \\ M_{P}=k_{Pt}\beta ,M_{Q}=k_{Qt}\beta \end{array}\right.$$

where *k_Pb_* and *k_Qb_* are the bending stiffness of the flexible hinges *P* and *Q*, and *k_Pt_* and *k_Qt_* are the torsion stiffness of the flexible hinges *P* and *Q*. Substituting Equation (38) into Equation (37), we can obtain

(39) $$\left\{\begin{array}{c} \beta =\frac{l_{9}}{k_{Pt}+k_{Qt}}F_{Px}\\ \varepsilon =\frac{1}{k_{Qb}}F_{Px} \end{array}\right.$$

Substituting Equation (39) into Equation (36), the stiffness of the transmission mechanism can be written as follows:

(40) $$k_{d}=\frac{k_{Qb}\left(k_{Pt}+k_{Qt}\right)}{l_{9}^{2}k_{Qb}+k_{Pt}+k_{Qt}}$$

### 3.4. Static Modeling of the Stage

The positioning stage can be regarded as a series combination of the amplification mechanism, the guiding mechanism, and the transmission mechanism. The guiding mechanisms and transmission mechanisms are both connected to the work platform, which is regarded as a parallel combination. The loading effect of the guiding and transmission mechanisms on the amplification mechanism is equivalent to an elastic load with the port stiffness of *K_load_*. Hence,

(41) $$K_{load}=k_{g}+2k_{d}$$

The two-stage amplification mechanism is a series combination of the bridge mechanism and the lever mechanism. By combining Equations (21) and (32), which represent the output stiffness of the bridge mechanism and the lever mechanism with load, respectively, the overall stiffness *K_amp_* of the amplification mechanism can be expressed as follows:

(42) $$K_{amp}=\frac{k_{b\_out}k_{L}}{k_{b\_out}+k_{L}}$$

According to reference [36], the amplification ratio of the overall compliant stage is expressed as follows:

(43) $$R_{stage}=\frac{K_{amp}}{K_{amp}+K_{load}}\lambda_{B}\lambda_{L}$$

### 3.5. Parameter Analysis

**Figure 5:**
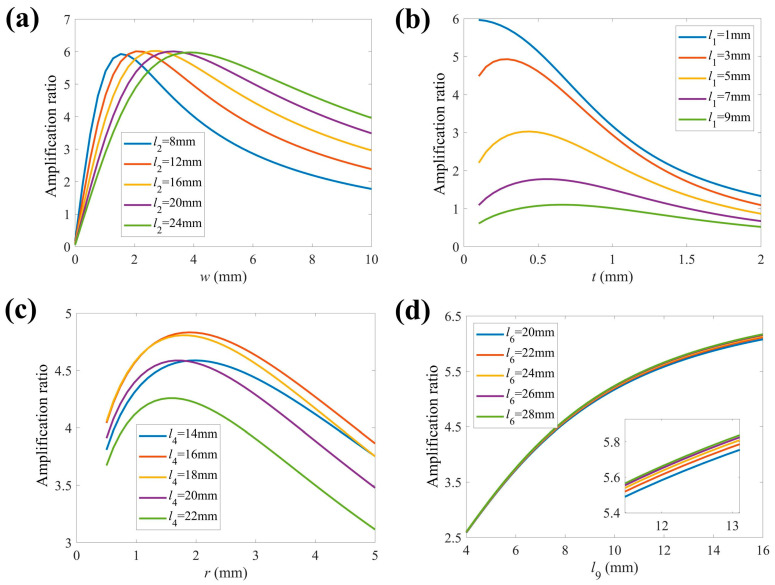
The effects of structural parameters on displacement amplification ratio. (**a**) *w* and *l*_2_, (**b**) *t* and *l*_1_, (**c**) *r* and *l*_4_, (**d**) *l*_9_ and *l*_6_.

To study the sensitivity of architectural parameters, we examined the influence of structural parameters on the amplification ratio, as shown in Figure 5a–d. The structural parameter values of the 2-DOF micro-positioning stage are listed in Table 1. Al7075 is selected as the manufacturing material of the 2-DOF stage, and its physical parameters are presented in Table 2.

From Figure 5a, one may observe that the amplification ratio first rises rapidly and then decreases slowly when *w* increases. The amplification ratio peaks with the increases in *l*_2_ and *w*. We can note that if *w* is within an acceptable range, the amplification ratio may vary non-monotonically with the increment of *l*_2_. In Figure 5b, one may observe that the amplification ratio increases first and the decreases slowly with *t* increasing. Meanwhile, the amplification ratio peaks at a fixed point with increment in *l*_1_. In Figure 5c, one may observe that the amplification ratio first increases and then declines slowly. It is worth noting that an increase *l*_4_ cannot lead to a thorough rise and reduction in the amplification ratio. As *l*_4_ increases, the amplification ratio reaches its peak value at *l*_4_ = 16 mm and then diminishes. In Figure 5d, one may observe that the amplification ratio initially rises rapidly and then grows slowly with increments in *l*_9_. Increasing *l*_6_ leads to a rise in the amplification ratio. Notably, when the value of *l*_9_ is small, the amplification ratio remains almost unchanged with variations in *l*_6_.

## 4. Dynamic Modeling Analysis

**Figure 6:**
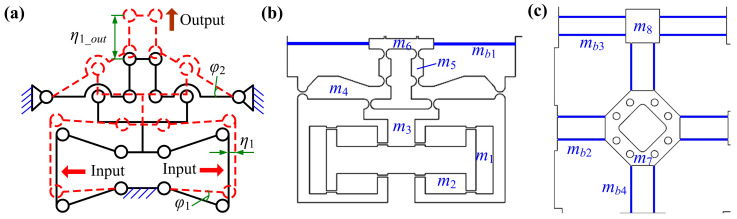
Dynamic modeling. (**a**) Geometric drawing based on pseudo-rigid-body method. Structure partition diagram of (**b**) the amplification mechanism and (**c**) the beam-based guiding mechanism.

In this section, the dynamic modeling of the compliant stage is presented for obtaining natural frequency via the pseudo-rigid-body method and Lagrange equation. To simplify the calculation, flexure hinges are regarded as rigid hinges without considering mass and axis center derivation based on the pseudo-rigid-body method. Additionally, due to tiny deformation of the stage under a small input displacement, each lever is regarded as an even rod for convenience of calculating the moment of inertia. The geometric drawings of the amplification mechanism and structure partition diagram of the stage are presented in Figure 6. The detailed mass parameter values of the 2-DOF stage are presented in Table 3. The vector of coordinates $\eta ={\left[\eta_{1},\eta_{2}\right]}^{T}$ is employed to represent the movements of the X- and Y-axes. The Lagrange equation is expressed as shown below the figure.

(44) $$\frac{d}{dt}\frac{\partial T}{\partial {\dot{\eta }}_{i}}-\frac{\partial T}{\partial \eta_{i}}+\frac{\partial V}{\partial \eta_{i}}=F_{i}$$

where *T* and *V* denote kinetic energy and potential energy existing within the mechanism, and where *F_i_* (*i* = 1, 2) is the x-axis and y-axis actuation force. Given the equivalent mass *M* and stiffness *K*, the dynamics equation for the conservative system is derived as follows:

(45) $$M\ddot{\eta }+K\eta =0$$

The total potential energy of the whole stage is $V=V_{\eta 1}+V_{\eta 2}$, where $V_{\eta 1}=V_{\eta 2}$. The potential energy in the *y*-direction output is derived as follows:

(46) $$V=\frac{1}{2}K_{stage}\eta_{1}^{2}$$

**Figure 8:**
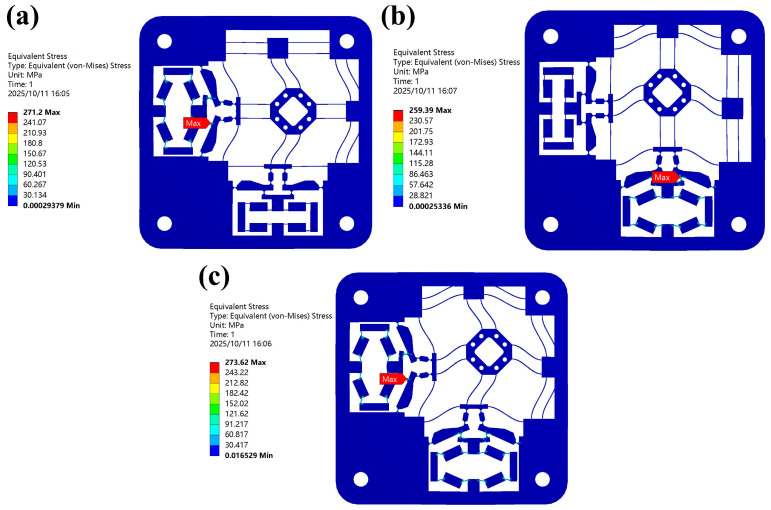
The von Mises stress simulation results (**a**) along the *x*-direction, (**b**) along the *y*-direction, (**c**) along both the *x*- and *y*-direction.

Similarly, for kinetic energy, we can obtain $T=T_{\eta 1}+T_{\eta 2}$, where $T_{\eta 1}=T_{\eta 2}$. As shown in Figure 8, the kinetic energy of the stage with *y*-direction output can be calculated as follows:

(47) $$\begin{array}{l} T=2\times \frac{1}{2}m_{1}{\dot{\eta }}_{1}^{2}+4\times \frac{1}{2}\frac{m_{2}\left(w^{2}+l_{2}^{2}\right)}{3}{\left(\frac{{\dot{\eta }}_{1}}{\sqrt{w^{2}+l_{2}^{2}}}\right)}^{2}+\frac{1}{2}m_{3}{\left(\lambda_{B}{\dot{\eta }}_{1}\right)}^{2}+2\times \frac{1}{2}\frac{m_{4}l_{5}^{2}}{3}{\left(\frac{\lambda_{B}{\dot{\eta }}_{1}}{l_{5}}\right)}^{2}+2\times \frac{1}{2}m_{5}{\left(R_{stage}{\dot{\eta }}_{1}\right)}^{2}\\ +\frac{1}{2}m_{6}{\left(R_{stage}{\dot{\eta }}_{1}\right)}^{2}+\frac{1}{2}m_{7}{\left(R_{stage}{\dot{\eta }}_{1}\right)}^{2}+\frac{1}{2}m_{8}{\left(R_{stage}{\dot{\eta }}_{1}\right)}^{2}+2\times \frac{1}{2}\frac{m_{b1}l_{6}^{2}}{3}{\left(\frac{R_{stage}{\dot{\eta }}_{1}}{l_{6}}\right)}^{2}+4\times \frac{1}{2}\frac{m_{b2}l_{7}^{2}}{3}{\left(\frac{R_{stage}{\dot{\eta }}_{1}}{l_{7}}\right)}^{2}\\ +4\times \frac{1}{2}\frac{m_{b3}l_{8}^{2}}{3}{\left(\frac{R_{stage}{\dot{\eta }}_{1}}{l_{8}}\right)}^{2}+4\times \frac{1}{2}m_{b4}{\left(R_{stage}{\dot{\eta }}_{1}\right)}^{2} \end{array}$$

Based on Equations (45)–(47), the equivalent mass *M* and stiffness *K* can be represented as follows:

(48) $$\begin{array}{l} M=2m_{1}+\frac{4m_{2}}{3}+m_{3}\lambda_{B}^{2}+\frac{2m_{4}\lambda_{B}^{2}}{3}+2m_{5}R_{stage}^{2}+m_{6}R_{stage}^{2}+m_{7}R_{stage}^{2}\\ +m_{8}R_{stage}^{2}+\frac{2m_{b1}R_{stage}^{2}}{3}+\frac{4m_{b2}R_{stage}^{2}}{3}+\frac{4m_{b3}R_{stage}^{2}}{3}+4m_{b4}R_{stage}^{2} \end{array}$$

(49) $$K=K_{stage}=\frac{K_{amp}K_{load}}{K_{amp}+K_{load}}$$

Therefore, the natural frequency of the stage can be obtained as follows:

(50) $$f=\frac{1}{2\pi }\sqrt{\frac{K}{M}}$$

## 5. Simulation Analysis

To validate the accuracy of the analytical model, the finite element (FE) simulation of the 2-DOF compliant stage using Workbench8.0 software is presented. Based on Section 3.5, considering the structural compactness and practical working environment, the architectural parameter values and selected materials of the stage are listed in Table 1 and Table 2. To improve calculation accuracy, the 2-DOF stage is meshed with 3027 elements and 20,468 nodes; the size of the unit node is 0.5 mm; a mesh convergence analysis was conducted to ensure the accuracy of the results. During the simulation, the four holes were fixed to restrict motion of the overall 2-DOF stage. Next, two lateral displacements were applied to the input ends of the two sides of the bridge mechanism, respectively. The output displacement was generated along the longitudinal direction of the output end. The multiple different input displacements were applied for calculating the amplification ratio to ensure accuracy of simulation results.

**Figure 7:**
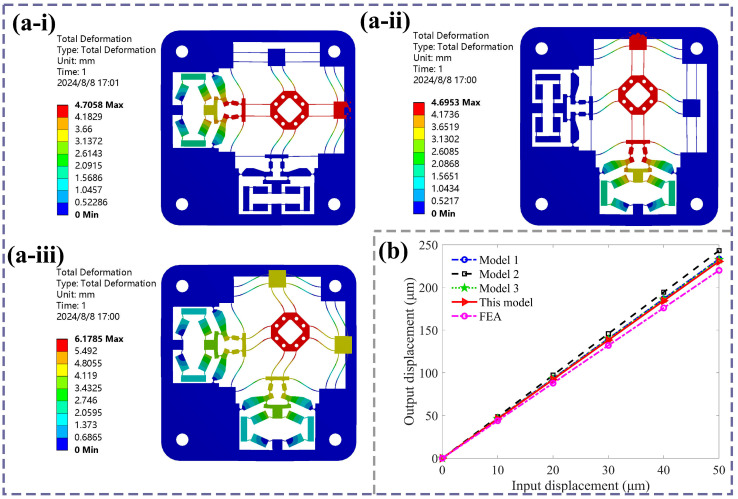
(**a**) The FE results of the positioning stage, (**i**) along the *x*-direction, (**ii**) along the *y*-direction, (**iii**) along both the *x*- and *y*-direction. (**b**) Comparison of existing analytical models and FE results.

The FE simulation contour of the 2-DOF positioning stage with output displacements along the *x*-direction, *y*-direction, and both *x*- and *y*-directions simultaneously are presented, as shown in Figure 7a. The output displacement simulation results are obtained by adding the input displacement at the *x*-direction input end of the bridge amplification mechanism. Meanwhile, to highlight the accuracy of this model, existing typical analytical models of bridge mechanisms from refs. [44,45,46] are calculated for comparison of the amplification ratios of the overall compliant stage. Figure 7b presents a comparison between existing analytical models and our simulation results of the output displacement. The different analytical amplification ratios and errors are presented in Table 4. It can be observed from Figure 7b that the amplification ratio trends of all analytical models matched well with that of the finite element results. The analytical amplification ratios are 4.67, 4.86, and 4.65. Among these, in references [45,46], the tensile stiffness and rotational stiffness of the flexible hinges had been considered comprehensively using geometric deformation and the elastic beam theory, resulting in a relatively accurate model. The theoretical model results in this work have a better consistency with simulation curves by combining the elastic beam theory and Castigliano’s second theorem compared to other existing analytical models. From Equation (43), the theoretical displacement amplification ratio is 4.61 with an error of 4.6%, while the simulation ratio is 4.40. The error is due to the following factors: the center of the flexible hinge has shifted during the stage’s motion; the elastic loading stiffness of the guiding mechanism may deviate from the analytical calculation value; and the simulation calculation’s accuracy is limited by computer function and condition settings.

The von Mises stress simulation results of the 2-DOF positioning stage along the *x*-direction, *y*-direction, and both *x*- and *y*-directions simultaneously are presented, as shown in Figure 8. We can observe the stress distribution in the 2-DOF stage in ANSYS under the input displacement of 50 μm in each direction. With input displacement in different directions, the max stress always occurs at circular hinge *I* (input end) in the lever mechanism, where the maximum von Mises stress (271.2, 259.4, and 273.6 MPa) is lower than the yield stress of Al7075 (503 MPa).

**Figure 9:**
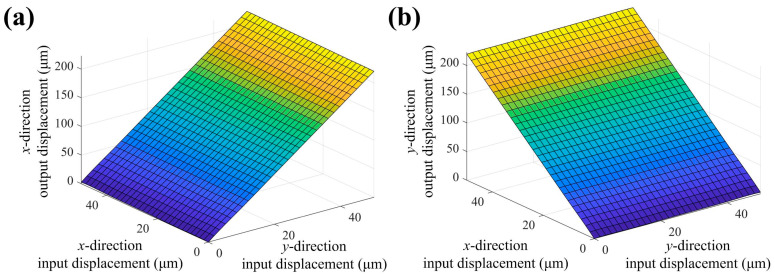
FEA results of output and different-direction input displacement. (**a**) x-direction output, (**b**) y-direction output.

To verify the motion accuracy of the 2-DOF stage, the output displacement for input displacements along different directions is calculated through FE simulation, as shown in Figure 9. It is worth noting that the input direction of the bridge-type amplification mechanism is perpendicular to its output direction. As shown in Figure 9a, the output displacement of the compliant stage along the y-direction rises linearly with the increase in the input displacement in the *x*-direction. Meanwhile, the output displacement in the *y*-direction is almost not affected by increases in input along the *y*-direction. Similarly, from Figure 9b, the same conclusion can be drawn. Thus, the 2-DOF compliant stage exhibits excellent decoupling performance and can achieve high-precision motion in specific directions.

**Figure 10:**
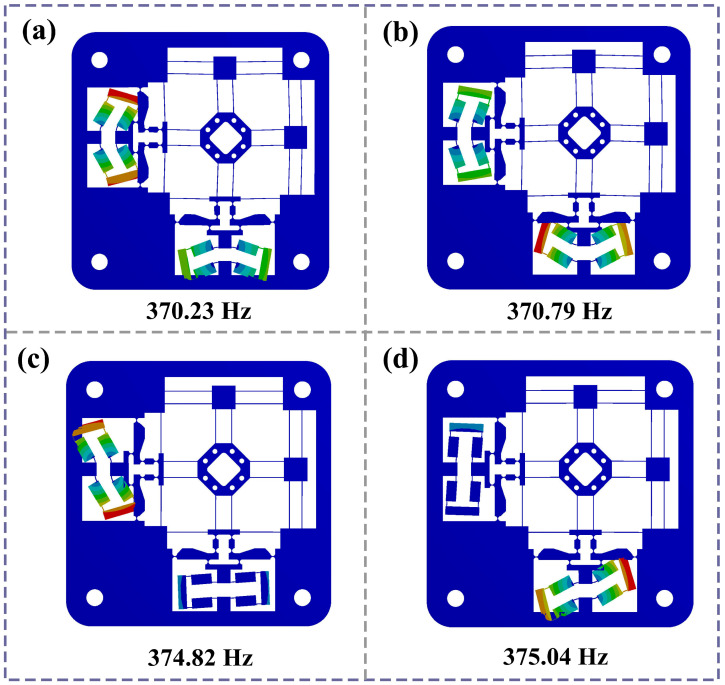
Mode simulation results. (**a**) The first order: 370.23 Hz; (**b**) the second order: 370.79 Hz; (**c**) the third order: 374.82 Hz; (**d**) the fourth order: 375.04 Hz.

To investigate the dynamic response characteristics of the compliant stage, modal analysis is conducted. The first four resonant modes, as well as their respective natural frequencies, are presented in Figure 10. One may observe that the compliant stage has a high natural frequency (370.23 Hz). The first four resonant modes are primarily in-plane warp motion of the two bridge amplification mechanisms, not the working platform. Based on dynamic modeling, the calculated resonant frequency (334.61 Hz) is compared with simulation result, as shown in Table 5. The error (9.7%) is due to the following factors: there is a difference between the deformation mode and the motion of the overall stage for analytical calculation; and only the rotation of the flexure hinge was analyzed without considering other factors, such as the displacement of the hinge center. The warp motions of the two bridge mechanisms are both obvious in the *xy* plane. As the frequency increases, the two bridge-type structures alternately generate the same type of twisting motion. The third mode occurs in the bridge mechanism with *y*-direction input, while the motion of the bridge mechanism with *x*-direction input is predominant in fourth mode. Therefore, the 2-DOF compliant stage has a high level of stability and exhibits favorable dynamic performance.

**Figure 11:**
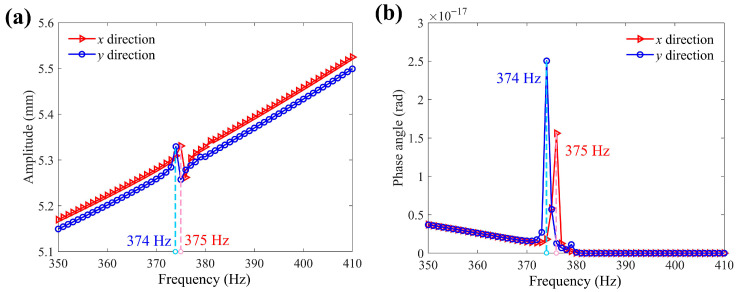
The frequency response simulation results. (**a**) Amplitude–frequency plot with *x*-direction and *y*-direction output; (**b**) phase–frequency plot with *x*-direction and *y*-direction output.

To investigate frequency response characteristics of the 2-DOF positioning stage, harmonic response simulation from 350 Hz to 410 Hz with 60 steps is conducted. The amplitude–frequency and phase–frequency plot with x-direction and y-direction output, respectively, are presented in Figure 11. The resonance frequencies of the 2-DOF positioning stage with *x* and *y*-direction output are 374 Hz and 375 Hz, respectively, approaching the first-order natural frequency. It can be observed that no matter in which direction the input displacement is applied, the resonance amplitude of the platform in the output direction is subtle. On the other hand, the phase angle of the positioning stage changes significantly at the resonance frequency, which is reflected in the resonant mode (in Figure 10).

## 6. Conclusions

The design and characterization of a 2-DOF micro-positioning stage for vibration-assisted polishing are presented in this paper. Based on Castigliano’s second theorem and elastic beam theory, the displacement amplification ratio and stiffness characteristics of the bridge–lever-type mechanisms, guiding beams, and transmission mechanisms are obtained. The dynamic modeling of the compliant stage is derived via the pseudo-rigid-body method and Lagrange equation. Finally, FE simulation analysis is conducted to validate the analytical model.

By means of reasonable arrangement of flexure elements and an optimized lever shape, the 2-DOF compliant stage can realize precise translational motion and large-stroke output. By comparing existing static models, we showed that this proposed model has a better agreement with simulation results. The analytical and simulation amplification ratios of the compliant stage are 4.61 and 4.40, respectively, which meets the working range requirements for providing vibration displacement. The stress analysis results demonstrate that the stage can work well in a normal operation environment. Additionally, the 2-DOF stage exhibits good motion-coupling characteristics. Modal analysis reveals that the positioning stage has a large resonant frequency, which is not affected by vibration from piezoelectric actuators. Harmonic response analysis shows that the resonance vibration of the working platform is subtle at the resonant frequency. In our future work, a practical prototype of the 2-DOF compliant stage will be fabricated, and the polishing system using the piezo-driven motion stage will also be tested to verify its effectiveness and reliability in precision polishing.

## Figures and Tables

**Table 1:** Architectural parameters.

Parameter	Value (mm)	Parameter	Value (mm)
*l*_0_	18.5	*b*	10
*l*_1_	3	*w*	4.5
*l*_2_	12	*t*_0_	5
*l*_3_	8	*t*	0.5
*l*_4_	19.75	*h*	6
*l*_5_	24.25	*r*	1.5
*l*_6_	24	*l*_7_	20
*l*_8_	28	*l*_9_	8

**Table 2:** Physical parameters of Al7075 alloy.

Young’s Modulus	Poisson’s Ratio	Yield Strength	Density
72 GPa	0.33	503 MPa	2810 kg/m^3^

**Table 3:** Mass parameters of the compliant stage.

Parameter	Value (kg)	Parameter	Value (kg)
*m*_1_	0.0028	*m*_7_	0.013
*m*_2_	0.002	*m*_8_	0.0055
*m*_3_	0.0037	*m_b_*_1_	0.0003
*m*_4_	0.0036	*m_b_*_2_	0.0002
*m*_5_	0.0005	*m_b_*_3_	0.0004
*m*_6_	0.0016	*m_b_*_4_	0.0002

**Table 4:** Comparison between analytical models and simulation results.

Method	Amplification Ratio	Error
Model 1 [44]	4.67	6.1%
Model 2 [45]	4.86	10.4%
Model 3 [46]	4.65	5.6%
This model	4.61	4.6%
FEA	4.40	/

**Table 5:** Comparison between analytical models and simulation results.

Resonant Frequency	Theoretical Value	Simulation Value	Error
*f*	334.61 Hz	370.23 Hz	9.7%

## Data Availability

The data presented in this study are available on request from the corresponding author.

## Nomenclature

*F*, *M*	Force and moment
*O*, *O*_1_, *A*, *B*, *C*, *D*, *E*	Ends of beams in bridge mechanism
*l*_0_, *t*_0_, *l*_2_, *h*, *l*_1_, *t*, *l*_3_, *w*	Length and width of the input beam; length and width of the connection beam; length of the output beam; length and width of the straight beam’s hinge; horizontal distance between the two hinges in bridge mechanism
$k_{l0}$, $k_{l1}$, $k_{l2}$, $k_{l3}$	Axial tensile stiffness of the input beam; beam flexible hinges; connection beam and output beam
$k_{\theta 0}$, $k_{\theta 1}$, $k_{\theta 2}$, $k_{\theta 3}$	Rotational stiffness of the input beam; beam flexible hinges; connection beam and output beam
*x_in_*, *x_out_*	Input and output displacements of the bridge mechanism
*k_b-in_*, *k_b_out_*, *λ_B_*	Input stiffness, output stiffness, and amplification ratio of the bridge mechanism
*H*, *I*, *J*	Flexible hinges in lever mechanism
*l*_4_, *l*_5_, *r*, *t*, *b*, *α*, *δ*	Horizontal distance between hinges *H* and *I*; horizontal distance between hinges *H* and *J*; circular radius; minimum width and thickness of the circular hinge; rotational angle of hinge *H*; and deviation displacement of center of hinge *H* in lever mechanism
*k_Hb_*, *k_Jb_*, *k_Ht_*, *k_It_*, *k_Jt_*	Bending stiffness of flexible hinges *H* and *J* and torsion stiffness of flexible hinges *H*, *I*, and *J*
*k_L_*, *λ_L_*	Stiffness and amplification ratio of the lever mechanism
*t_s_*, *l_s_*, *k_s_*,	Thickness, length, and bending stiffness of a single beam
*l*_6_, *l*_7_, *l*_8_	Length of beams in guiding mechanism
*k_g_*	Stiffness of guiding mechanism
*P*, *Q*	Flexible hinges in transmitting mechanism
*l*_9_	Vertical distance between hinges *P* and *Q*
*β*, *ε*	Rotational angle and axial drift displacement of hinge *Q*
*k_Pb_*, *k_Qb_*, *k_Pt_*, *k_Qt_*	Bending stiffness of flexible hinges *P* and *Q* and torsion stiffness of flexible hinges *P* and *Q*
*k_d_*	Stiffness of the transmission mechanism
*K_load_*, *K_amp_*, *K_stage_*	Stiffness of elastic load, overall stiffness of the amplification mechanism, and amplification ratio of the compliant stage
*η_i_*, *φ_i_*, *T*, *V*, *m_i_*, *f*	Movement, rotational angle, kinetic energy, potential energy, mass, and frequency
*E*, *b*	Young’s modulus and out-of-plane width of the compliant stage
